# Fructilactobacillus frigidiflavus sp. nov., a pigmented species, and Levilactobacillus lettrarii sp. nov., a propionate-producing species isolated from sourdough

**DOI:** 10.1099/ijsem.0.006726

**Published:** 2025-03-20

**Authors:** Vi D. Pham, Michael G. Gänzle

**Affiliations:** 1 Department of Agricultural, Food and Nutritional Science, University of Alberta, Edmonton, Canada

**Keywords:** 1,2-propanediol, C30 carotenoid, *Fructilactobacillus*, *Lactobacillus*, *Levilactobacillus*, pigmentation, propionic acid, sourdough

## Abstract

The sourdough isolates FUA3702, FUA3912 and FUA3913^T^, as well as FUA3695^T^ and FUA3914, could not be identified to known species of the *Lactobacillaceae*. The 16S rRNA gene sequences of FUA3702 and FUA3913, FUA3695 and FUA3914 were>99% identical to *Fructilactobacillus sanfranciscensis* and *Levilactobacillus lanxiensis*, respectively. The average nucleotide identity (ANI) and digital DNA–DNA hybridization (dDDH) values of strain FUA3913^T^ when compared to *Fl. sanfranciscensis* were 83.67 and 26.60%, respectively. In addition, strains FUA3702, FUA3912 and FUA3913^T^ produce different levels of a yellow C30 carotenoid, but pigmentation has not been described in *Fl. sanfranciscensis*. The ANI and dDDH values of FUA3695^T^ and FUA3914 when compared to *Lv. langxiensis* were 95.22 and 61.20%, respectively. In addition, FUA3695 and FUA3914 convert lactate to 1,2-propanediol and further to propionate. The conversion of lactate to propionate by a single strain has not been documented for any of the species in the *Lactobacillaceae*. Based on the genomic and physiological characteristics, we proposed the novel species *Fructilactobacillus frigidiflavus* sp. nov. FUA3913^T^ (=DSM 118650^T^=LMG 33758^T^) and *Levilactobacillus lettrarii* sp. nov. FUA3695^T^ (=DSM 118651^T^=LMG 33759^T^).

## Introduction

Species of the *Lactobacillaceae* family are the most common isolates from sourdough. Up to 111 different species of lactobacilli have been identified in sourdough fermentations [1]. The scientific and commercial interest in sourdough has been growing. Between 2014 and 2023, the number of publications that were retrieved with Google Scholar with the keywords ‘sourdough isolates’ increased from 981 to more than 2,500 entries. Although a substantial number of sourdough isolates were assigned to novel species in the past decades, the last species new description with sourdough isolates was published in 2013 [2] or with isolates obtained in 2013 [3], indicating that the microbial communities in sourdoughs have been comprehensively characterized.

At the household level, in artisanal bakeries and in industrial baking, sourdough is generally maintained by continuous back-slopping [4]. The propagation conditions differ substantially depending on whether the purpose of sourdough fermentation is leavening of the dough, acidification of the dough or its use as a baking improver [4,6]. Sourdoughs used as the sole leavening agent maintain micro-organisms continuously in a metabolically active state; the sourdough propagation includes frequent (2–4 times per day) refreshments and fermentation at ambient (15–25 °C) temperature. These sourdoughs generally are populated by *Fructilactobacillus sanfranciscensis* and *Kazachstania humilis* [5,7]. Industrial processes that aim to achieve dough acidification are propagated with long fermentation time (1–7 days) at a temperature of 30–45 °C. In most of these sourdoughs, fermentation conditions are selected for acid-resistant and thermophilic *Lactobacillus* and *Limosilactobacillus* species [5,7, 8]. Sourdoughs used as baking improver are often inoculated with starter cultures [4]. In the last few years, an increasing number of ‘amateur bakers’ propagate sourdough in the household for extensive baking once every few weeks [6]. Owing to the greater diversity of sourdough propagation schemes, sourdoughs that are maintained at the household level harbour a greater diversity of lactobacilli. *Levilactobacillus*, *Companilactobacillus*, *Lactiplantibacillus* and *Pediococcus* species were frequently identified in these sourdoughs [9].

The isolates described in this study were obtained from a sourdough that is used for production of both bread and pasta [10]. FUA3702 and FUA3695 were isolated from the same batch of sourdough. FUA3912, FUA3913 and FUA3914 were isolated from the same sourdough a few years later. The propagation scheme is based on fermentation steps at 4–8 °C for 7 days [11]. Comparable propagation schemes are not documented in the scientific literature [5,7, 12]. The exceptional sourdough propagation scheme resulted in an equally exceptional composition of micro-organisms [11]. Several of the isolates could not be assigned to known species. It was therefore the aim of this study to determine genomic and physiological characteristics of these isolates and to assess whether these isolates represent novel species in the *Lactobacillaceae*.

## Bacterial isolation, genome sequencing and analyses

Sourdough was homogenized in sterile 1% peptone and 0.9% NaCl, and serial tenfold dilutions were plated onto modified de Man–Rogosa–Sharpe 6 (MRS6) [13] and MRS5 [14] agar supplemented with 100 mgl^−1^ of cycloheximide to inhibit growth of yeasts. mMRS6 has the same composition as mMRS5, but the addition of the vitamin mix and sodium acetate is replaced by the addition of 10% of malt extract. These media support the growth of a wide range of sourdough lactobacilli [15,16]. Single colonies were purified by repetitive dilution streaks on the corresponding agar plates, and purified isolates were stored with 30% glycerol at −80 °C. DNA isolation, genome sequencing on the Oxford Nanopore platform with 100–200-fold coverage and genome assembly using high-quality reads were described previously [11].

## Phylogeny of isolates

The 16S rRNA genes were Sanger sequenced and later confirmed by genome sequencing. The 16S rRNA genes of FUA3702, FUA3912 and FUA3913, as well as FUA3695 and FUA3914, are 99.1 and 99.94% identical to those of *Fl. sanfranciscensis* and *Levilactobacillus lanxiensis*, respectively, i.e. above the threshold of 98.65% below which 16S rRNA gene sequences inform on species differentiation [17]. The 16S rRNA genes of FUA3702, FUA3912 and FUA3913 are also closely related to *Fructilactobacillus* spp. isolated from ants [18]. These isolates from ants were identified as *Fl. sanfranciscensis*, but their 16S rRNA genes are only 99.26% identical to *Fl. sanfranciscensis* and 99.36% identical to FUA3702, FUA3912 and FUA3913. Phylogenetic trees were calculated with 16S rRNA gene sequences of all type strains of fructilactobacilli and levilactobacilli. The three isolates each of *Fructilactobacillus* spp. and *Levilactobacillus* spp. form a separate clade in the phylogenetic trees of all species in the two genera (Figs S1 and S2, available in the online Supplementary Material).

**Fig. 1.**
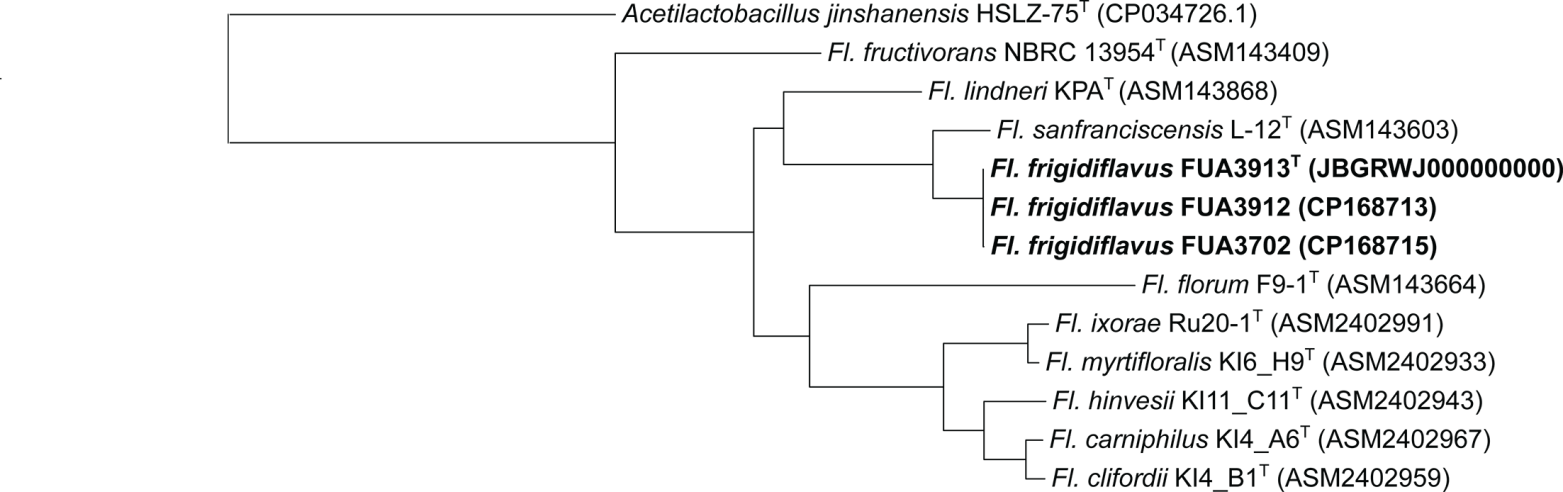
Phylogeny of FUA3702, FUA3912, FUA3913 and other ten species of *Fructilactobacillus* with the closest related species *A. jinshanensis* as an outgroup. The maximum-likelihood tree was constructed from 559 single-copy orthologues. GenBank accession numbers of the genomes are in parentheses. Superscript T indicates type strain.

**Fig. 2.**
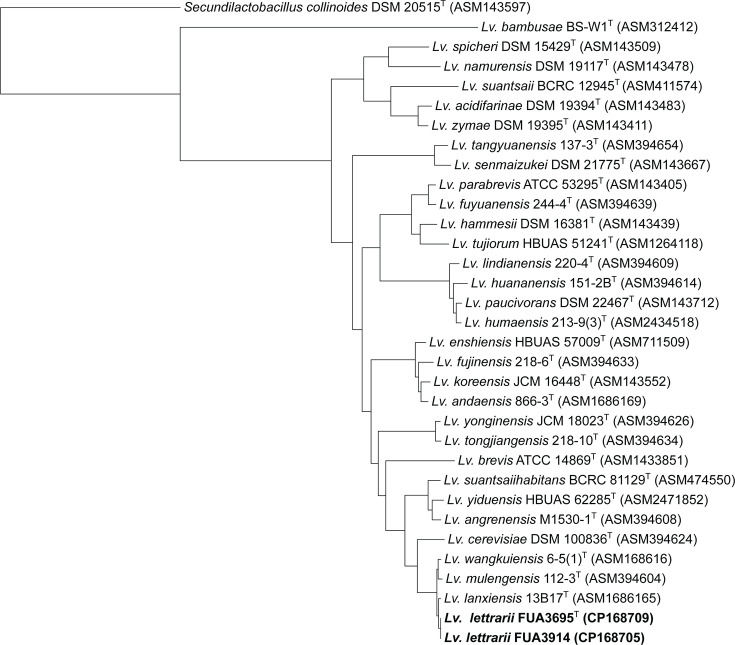
Phylogeny of FUA3695, FUA3914 and other known species of *Levilactobacillus* with the closest related species *Se. collinoides* as an outgroup. The maximum-likelihood tree was constructed from 815 single-copy orthologues. Assembly accession numbers of the genomes are in parentheses. Superscript T indicates type strain.

To infer the phylogeny of type species in the two genera, single-copy orthologues of type strains of the genera *Fructilactobacillus* and *Levilactobacillus* were determined by OrthoFinder and aligned using MAFFT [19,20]. The alignment was subjected to RAxML-NG for phylogenetic inference using the substitution model suggested by ModelTest-NG [21,22]. The closest genomes of the *Fructilactobacillus* and *Levilactobacillus*, *Acetilactobacillus jinshanensis* and *Secundilactobacillus collinoides*, respectively, were used as an outgroup. *Fructilactobacillus frigidiflavus* is most closely related to *Fl. sanfranciscensis* (Figs 1 and S1), and *Levilactobacillus lettrarii* is most closely related to *Lv. lanxiensis* (Fig. 2).

## Genome features and physiology of *Fructilactobacillus* spp. FUA3702, FUA3912 and FUA3913

The average nucleotide identity (ANI) values were calculated using orthology from OrthoANI [23]. The ANI values of *Fructilactobacillus* spp. FUA3702, FUA3912 and FUA3913 when compared to *Fl. sanfranciscensis* are 83.67–83.93%, below the established threshold of 95–96% for species delineation (Table 1) [24]. The digital DNA–DNA hybridization (dDDH) values were calculated as described [25]. The dDDH values of *Fructilactobacillus* spp. FUA3702, FUA3912 and FUA3913 in comparison to *Fl. sanfranciscensis* are 26.60%, also below the threshold of 70% to differentiate species (Table 1). The genome size of three strains is ~1.6 Mb (Table S1), which is substantially larger than the 1.23 Mb genome of *Fl. sanfranciscensis*. The G+C content of the three strains is ~37 mol% (Table S1), which is higher than those of *Fl. sanfranciscensis* and *Fructilactobacillus lindneri* (34–35 mol%) but in the range of values observed for other *Fructilactobacillus* sp. (34–47 mol%).

**Table 1.** dDDH values and ANI values (in parentheses) (%) of *Fructilactobacillus* spp. FUA3702, FUA3912 and FUA3913 and *Levilactobacillus* spp. FUA3695 and FUA3914 in comparison to the type strain of the closest related species

	FUA3912	FUA3913^T^	*Fl. sanfranciscensis* L-12^T^
FUA3702	99.80 (99.91)	99.80 (99.91)	26.60 (83.93)
FUA3912		100 (99.95)	26.60 (83.85)
FUA3913			26.60 (83.67)
	**FUA3695** ^**T**^		***Lv. lanxiensis* 13B17** ^**T**^
FUA3914	98.29 (100)		61.20 (95.39)
FUA3695			61.40 (95.32)

**Fig. 3.**
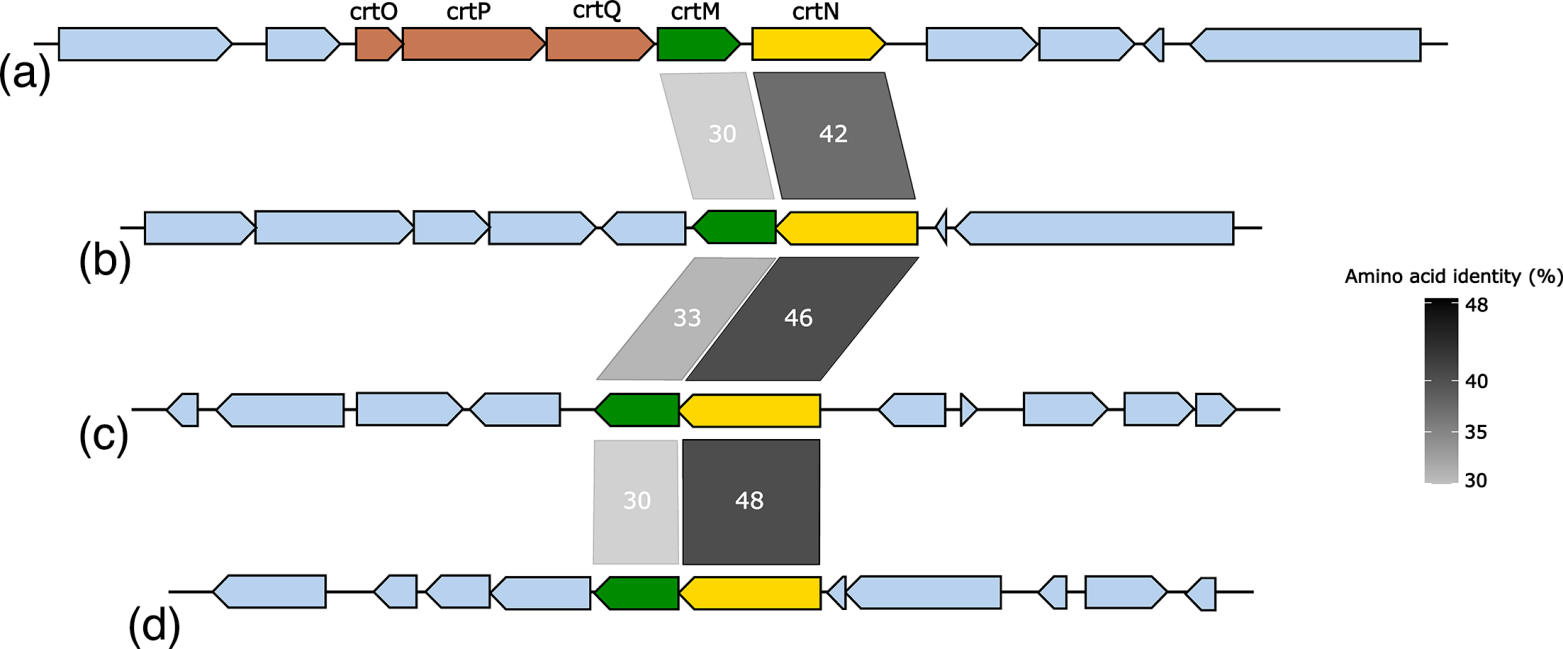
Comparison of the crtMN operon responsible for the synthesis of the yellow-pigmented C_30_ carotenoid, 4,4’-diaponeurosporene in *St. aureus* CN1 (a), *Fructilactobacillus* spp. FUA3913 (b), *Lp. plantarum* WCFS1 (c) and *L. mesenteroides* ATCC8293 (d). *St. aureus* possesses a full operon *crtOPQMN* and can further produce staphyloxanthin. Numbers and grey scale bar represent amino acid identity.

**Fig. 4.**
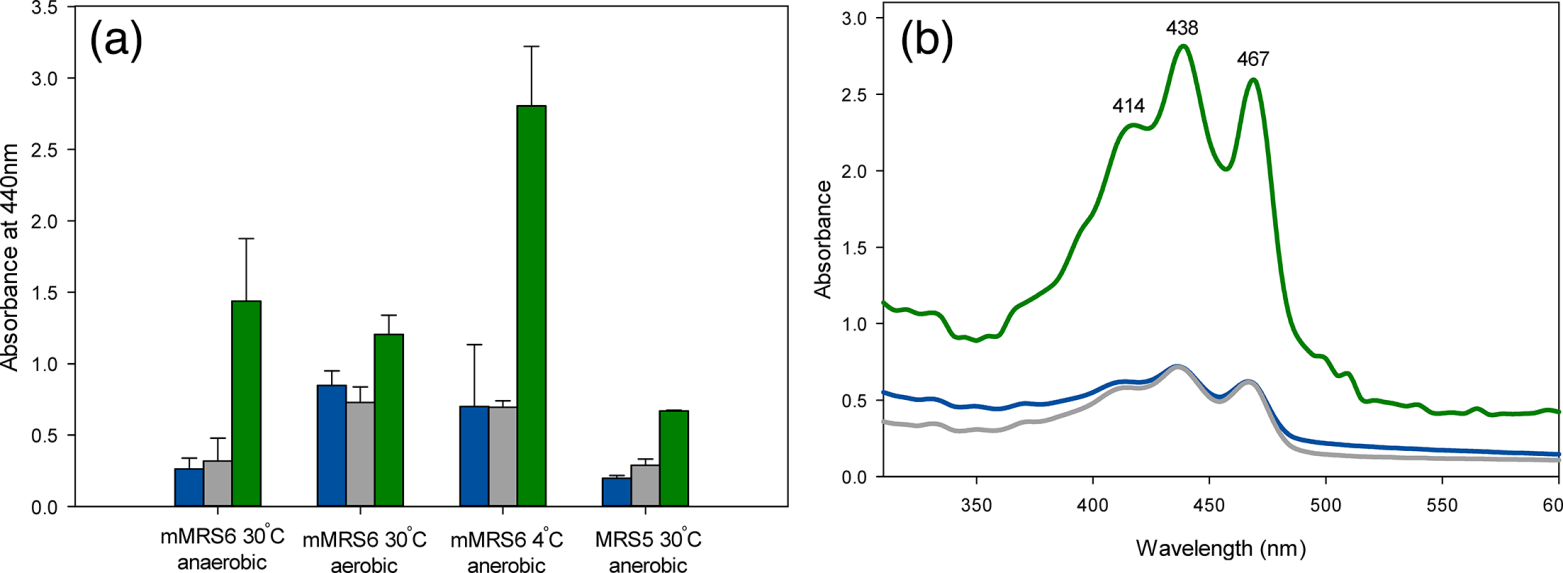
Absorbance of carotenoids extracted from cells of *Fructilactobacillus* spp. FUA3702 (blue), FUA3912 (grey) and FUA3913 (green) at maximum absorbance of 440 nm (a) and a full spectrum (b). Subcultures of cells were incubated for 2 days in mMRS media at the indicated conditions. Cells were washed with an equal volume of sterile water prior to freeze-drying. Pigments were extracted from equal dried cell mass and dilution as described [43]. Data are shown as mean, and the error bar represents the sd of three independent replicates. Corresponding extracts of *Fl. sanfranciscensis* L-12 did not show any absorbance at 350 nm or higher.

Colonies of *Fructilactobacillus* spp. FUA3702, FUA3912 and FUA3913 appeared yellow on agar plates; therefore, we searched for the genes encoding for the diapophytoene synthase CrtM and the diapophytoene saturase CrtN. These enzymes synthesize the yellow-pigmented C30 carotenoid 4,4’-diaponeurosporene in *Lactiplantibacillus plantarum* and *Leuconostoc mesenteroides* [26,27]. These genes are also present in the *crtOPQMN* operon that synthesizes staphyloxanthin in *Staphylococcus aureus* [28]. The genomes of all three strains code for CrtM with 30 and 33% identity to CrtM of *St. aureus* and *Lp. plantarum*, respectively (Fig. 3). The gene coding for CrtN is adjacent to CrtM, and the amino acid sequence is 42 and 46% identical to CrtN of *St. aureus* and *Lp. plantarum*, respectively (Fig. 3). The absorbance spectrum of the yellow pigments extracted from bacterial biomass of *Fructilactobacillus* spp. FUA3702, FUA3912 and FUA3913 was identical to the absorbance spectrum of 4,4’-diaponeurosporene in *Lp. plantarum* [26] (Fig. 4a) with peaks at 414, 438 and 467 nm. As the CrtM and CrtN sequences of the three isolates and the absorbance spectra of the pigments are identical, all three strains likely produce 4,4’-diaponeurosporene. Pigmentation in *Lactobacillaceae* is not well documented, but a recent finding identified that CrtM and CrtN were widespread in genera associated with pollinating insects and plants [29]. The genus *Fructilactobacillus* was proposed to have an insect/flower-associated lifestyle [30], and few strains of *Fl. sanfranciscensis* carry CrtM/N [29].

The level of pigmentation in the three strains was impacted by the growth conditions (Fig. 4b). Cold temperature enhanced pigmentation of stationary-phase cultures in all three strains; aeration and the growth medium impacted pigmentation in a strain-specific manner. *Fructilactobacillus* spp. FUA3913 produced a higher level of pigments than *Fructilactobacillus* FUA3702 and FUA3912 regardless of condition, with the highest level of pigmentation observed after incubation at 4 °C. Aerobic conditions enhanced the synthesis of pigments in *Lp. plantarum*, but the effect of cold temperature on pigmentation has not been reported [31].

**Fig. 5.**
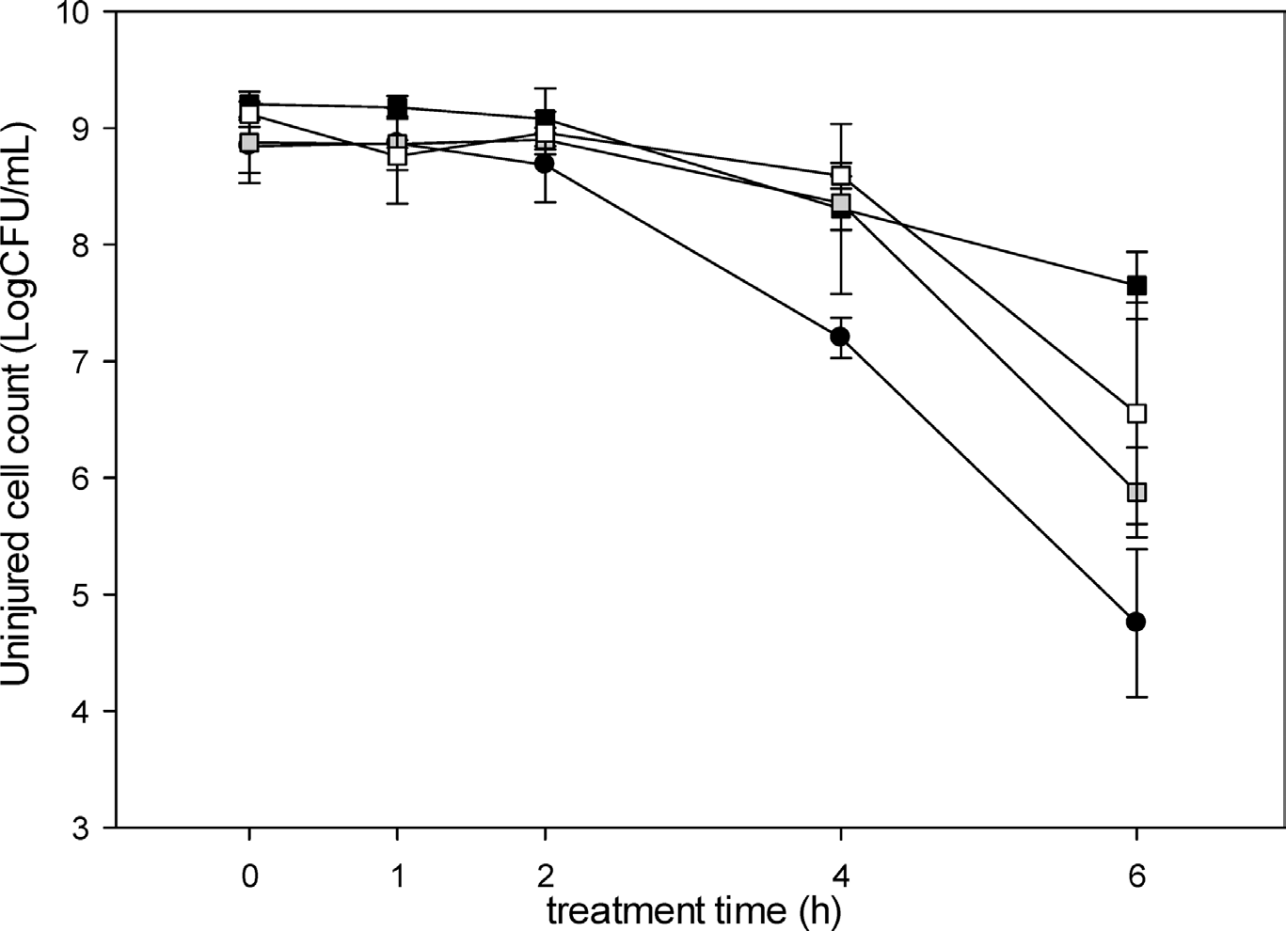
Hydrogen peroxide resistance of *Fl. frigidiflavus* (squares) compared to *Fl. sanfranciscensis* L-12 (circle). Black, grey and white squares represent *Fructilactobacillus* FUA3912, FUA3913 and FUA3702, respectively. Briefly, 1 ml of overnight culture was washed with an equal volume of sterile water and incubated with 7 mM hydrogen peroxide. Injured cells are pinpoint colonies compared to the untreated cells. Data and error bar are shown as the mean and sd of three independent replications.

Pigmentation protects bacteria against environmental stresses [29]; therefore, we compared the resistance to oxidative stress of the pigmented *Fructilactobacillus* spp. FUA3702, FUA3912 and FUA3913 to their closest non-pigmented relative, *Fl. sanfranciscensis* L-12. Cells were treated with 7 mM of hydrogen peroxide in sterile water and cell counts were determined over 6 h of treatment. Uninjured cells were differentiated from sublethally injured cells, as the latter formed only pinpoint colonies, and the cell count of uninjured cells is shown in Fig. 5. After 4 h, uninjured cell counts of *Fl. sanfranciscensis* were ~1.2 log c.f.u. lower than that of the pigmented strains (Fig. 5). After 6 h, cell counts of all strains decreased, but pigmented fructilactobacilli maintained higher cell counts of uninjured cells. The experiment did not include a water control to account for osmotic stress, but C30 carotenoids producing *Lp. plantarum* survive aeration and up to 6 mM of hydrogen peroxide [31,33]. Pigmentation in *Fructilactobacillus* spp. FUA3702, FUA3912 and FUA3913 thus likely increased resistance to oxidative stress.

After 2 days of incubation on mMRS6 agar, colonies of strain FUA3702 were 2.1 mm in diameter, circular with rough edges and light yellow. Colonies of strain FUA3912 were 1.3 mm, smooth, circular and light yellow. Colonies of strain FUA3913 were 0.9 mm in diameter, smooth, circular and vivid yellow. Cells were 1.23–2.11×0.62–0.94 µm (FUA3702), 1.2–4.32×0.82–1.28 µm (FUA3912) and 1.9–2.38×0.58–0.82 µm (FUA3913). Growth is observed at 4 and 45 °C and at pH 3.5–8.5. Carbohydrate metabolism was determined using the API 50 CH/CHL system (bioMerieux, France). Of 49 carbohydrates, only galactose and glucose were metabolized by *Fructilactobacillus* spp. FUA3702, FUA3912 and FUA3913 (Table 2). ANI, dDDH, pigmentation, temperature range of growth and carbohydrate metabolism differentiate the strains from their closest relative, *Fl. sanfranciscensis*, and justify the proposal of a new species, for which the name * Fl. frigidiflavus* is proposed.

**Table 2.** Carbohydrate metabolism of *Fructilactobacillus* spp., *Fl. sanfranciscensis*, *Levilactobacillus* spp. and *Lv. lanxiensis* determined by API 50 CH/CHL system Only metabolized carbohydrates are shown.

	*Fructilactobacillus* spp.			*Fl. sanfranciscensis*	*Levilactobacillus* spp.		*Lv. lanxiensis*
	FUA3702	FUA3912	FUA3913^T^	L-12^T^	FUA3695^T^	FUA3914	13B17^T^
d-Ribose	−	−	−	−	+	−	+
d-Xylose	−	−	−	−	+	+	+
d-Galactose	+	+	+	−	+	+	+
d-Glucose	+	+	+	−	+	+	+
d-Fructose	−	−	−	−	+	+	+
*N*-Acetylglucosamine	−	−	−	−	+	+	+
d-Maltose	−	−	−	+	+	+	+
d-Mannose	−	−	−	−	−	−	−
Melibiose	−	−	−	−	+	−	+
Sucrose	−	−	−	−	−	−	−
d-Raffinose	−	−	−	−	−	−	−
Turanose	−	−	−	−	−	−	−
Potassium gluconate	−	−	−	−	+	+	+

## Genome features and physiology of *Levilactobacillus* spp. FUA3695 and FUA3914

**Fig. 6.**
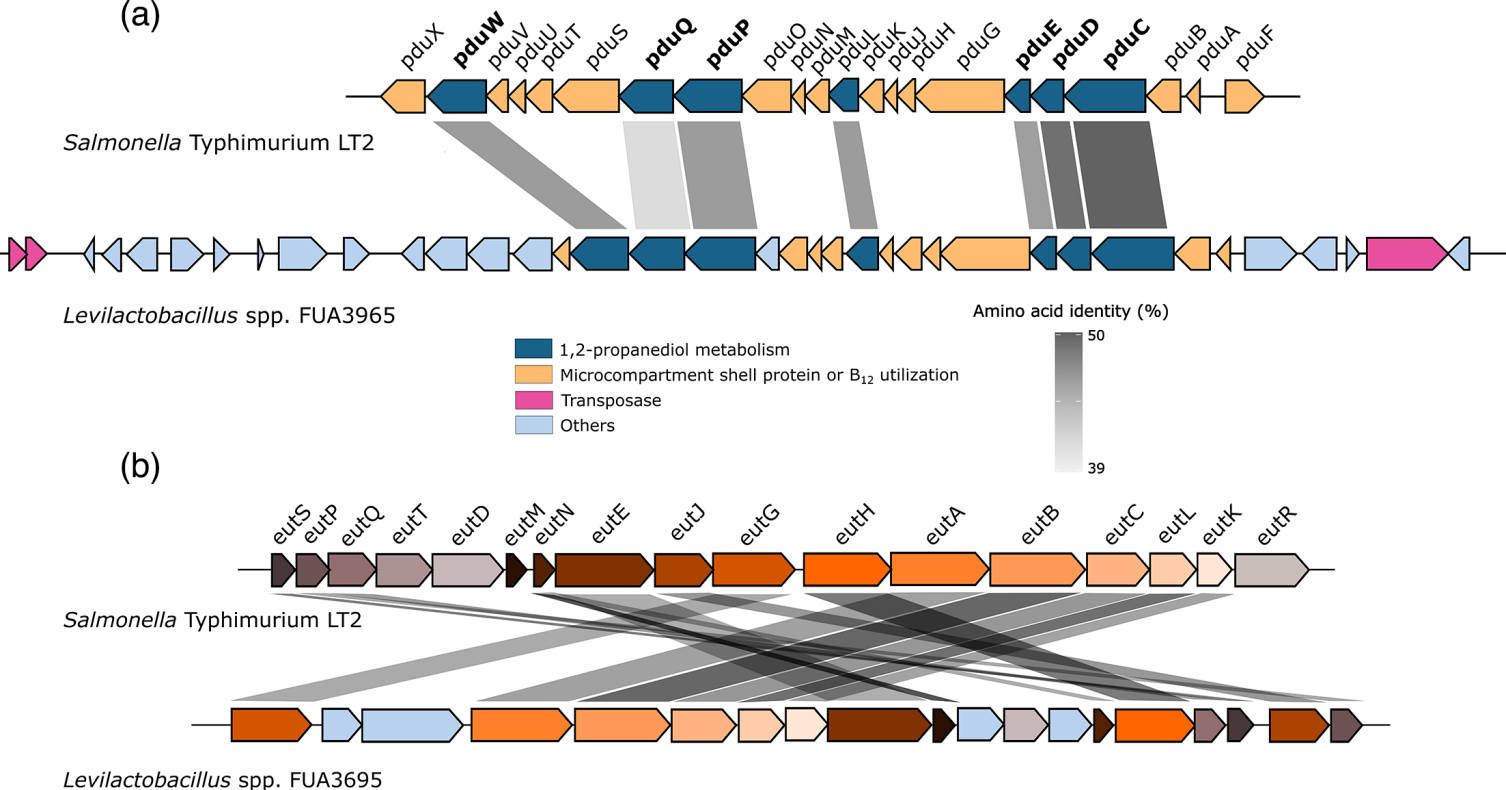
Comparative genomic regions of the *pdu* operon (a) and the *eut* operon (b) between *Levilactobacillus* spp. FUA3695 and *S. enterica* Typhimurium LT2 (GenBank accession no. AE006468.2). Grey scale bar represents amino acid identity. In the *pdu* operon, bold texts indicate that enzymes are functional, with results shown in Table 3. Transposases at both ends of the operon suggest the organisms might have acquired the operon via horizontal transfer. The *eut* operon of *Levilactobacillus* spp. FUA3695 only lacks two genes from the full operon of *Salmonella* with > 30% amino acid identity.

The ANI values of *Levilactobacillus* spp. FUA3695 and FUA3914 when compared to *Lv. lanxiensis* are 95.32–95.39% (Table 1). The respective dDDH values are 61.20–61.40%, below the threshold for speciation [34]. The genome size and the G+C content of the three strains are 3 Mb and 50 mol%, which are comparable to *Lv. lanxiensis* 13B17 (Table S1). The genomes of *Levilactobacillus* spp. FUA3695 and FUA3914 encode for the *pdu* operon that includes genes for proteins of microcompartment and the metabolic enzymes for conversion of 1,2-propanediol to propanol and propionate (Fig. 6a). The *pdu* operon and 1,2-propanediol metabolism are reported in *Salmonella enterica* Typhimurium [35], *Limosilactobacillus reuteri* [36], *Lentilactobacillus buchneri* [37], *Loigolactobacillus coryniformis* [38] and *Se. collinoides* [39], but microcompartments were only described in *Salmonella* and *Lm. reuteri*. In contrast to other lactobacilli, *Levilactobacillus* spp. FUA3695 and FUA3914 lack the *cob* operon, i.e. they are unable to synthesize cobalamin. Whether the *pdu* operon is present and functional in *Lv. lanxiensis* 13B17 was not reported [40]. The genomes of *Levilactobacillus* spp. FUA3695 and FUA3914 also encode for the *eut* operon that degrades ethanolamine but is not present in the genome of *Lv. lanxiensis* 13B17. Degradation of ethanolamine was described in *Furfurilactobacillus rossiae* as a differentiating feature to *Furfurilactobacillus milii* [3,41]. The enzymes encoded by the *eut* operon in *Levilactobacillus* spp. FUA3695 and FUA3914 are 30–67% identical to those of *S. enterica* Typhimurium (Fig. 6b) [3,41]. The *eut* operon is often present as the *pdu-cob-hem-eut* cluster in *Salmonella* [42].

The genomes of *Levilactobacillus* spp. FUA3695 and FUA3914 additionally encode sorbitol dehydrogenase, which converts sorbitol to fructose, and lactaldehyde dehydrogenase, which converts lactate to 1,2-propanediol. Accordingly, 1,2-propanediol was detected in culture supernatants of *Levilactobacillus* spp. FUA3695 and FUA3914 after 7 days of incubation, but sorbitol was not converted (Table 3). 1,2-propanediol was subsequently converted to propionate after 14 days (Table 3). The conversion of lactate to propanediol and propionate has been described for co-cultures of *Le. buchneri* and *Lentilactobacillus diolivorans* [37], but the same conversion by a single strain has not been reported to date.

**Table 3.** Production and conversion of 1,2-propanediol and production of propionate of *Levilactobacillus* spp. FUA3695 and FUA3914 The strains were grown in mMRS6, mMRS6 supplemented with 50 mM of lactate and mMRS6 supplemented with 50 mM of sorbitol at 30 °C for 7 days and 14 days. Metabolites (mM) were extracted and quantified using HPLC as described [37]. Negative values indicate consumption. Data are shown as mean and sd of three independent experiments.

		Lactate	Acetate	1,2 POH	Propionate	Maltose	Glucose	Fructose	Ethanol	Mannitol
**mMRS6**										
**7 days**	FUA3695	73.8±46.1	28.0±13.0	6.4±5.5	–	−37.0±0.1	−39.9±0.9	−38.6±1.2	40.4±20.4	14.9±13.8
	FUA3914	48.1±10.2	22.0±3.3	2.4±0.0	–	−36.9±0.1	−34.0±7.2	−35.2±5.7	24.6±0.7	13.3±11.0
**14 days**	FUA3695	66.6±48.2	30.4±6.9	4.3±4.6	6.3±0.1	−37.2±0.1	−51.7±0.1	−38.4±1.3	36.8±17.3	14.9±13.6
	FUA3914	46.7±18.5	22.1±2.1	1.1±0.0	5.9±1.3	−37.1±0.1	−40.3±1.5	−37.2±3.5	23.7±2.6	13.1±11.4
**mMRS6+50 mM lactate**										
**7 days**	FUA3695	56.1±4.0	8.6±3.6	–	–	0.0±0.5	−3.8±4.1	−5.1±5.0	1.2±0.3	4.1±4.7
	FUA3914	60.4±7.2	9.4±4.2	–	–	1.7±1.1	−0.3±1.7	−3.9±6.0	1.7±0.4	5.1±6.7
**14 days**	FUA3695	60.0±4.0	6.6±6.8	–	5.8±0.4	1.4±0.7	−3.7±2.7	−7.2±3.2	1.1±0.1	3.6±3.9
	FUA3914	60.0±5.9	9.5±1.8	–	5.7±0.0	2.4±0.9	1.4±1.5	−2.9±4.2	0.7±0.5	2.8±4.1
**mMRS6+50 mM sorbitol**										
**7 days**	FUA3695	78.0±33.0	26.8±8.5	4.8±3.3	–	−17.4±14.4	−35.0±9.2	−34.6±2.7	37.1±18.1	23.9±2.4
	FUA3914	93.2±0.5	27.7±1.1	4.5±0.5	–	−21.1±0.9	−41.3±0.3	−35.1±0.5	46.6±0.3	22.6±0.4
**14 days**	FUA3695	91.1±24.0	29.5±6.2	4.5±3.5	5.6±1.0	−20.6±15.2	−51.3±1.2	−35.4±2.1	39.7±14.0	25.1±0.4
	FUA3914	75.9±19.9	21.7±5.1	2.1±1.4	5.3±0.4	−15.1±13.9	−41.8±3.1	−34.8±0.1	34.3±9.7	22.9±0.0

*Levilactobacillus* spp. FUA3695 and FUA3914 metabolize xylose, galactose, glucose, fructose, *N*-acetylglucosamine, maltose and potassium gluconate. *Levilactobacillus* spp. FUA3695 also produced acid from ribose and melibiose (Table 2). *Levilactobacillus* spp. FUA3695 and FUA3914 are the first lactobacilli for which conversion of lactate via 1,2-propanediol to propionate has been documented; in addition, the presence of the *eut* operon also differentiates the strains from * Lv. lanxiensis*. The dDDH values and the differentiating metabolic features differentiate the strains from their closest relative, *Lv. lanxiensis*, and justify the proposal of a new species, for which the name *Lv. lettrarii* is proposed.

## Description of *Fructilactobacillus frigidiflavus* sp. nov.

*Fructilactobacillus frigidiflavus* (fri.gid.i.fla’vus. L. masc. adj. *frigidus*, cold; L. masc. adj. *flavus*, yellow; N.L. masc. adj. *frigidiflavus*, yellow in the cold).

Cells are Gram-stain positive, non-spore forming, rod-shaped, ~1.2–4.3 µm long and 0.58–1.3 µm wide and occur as single cells or short chains. Colonies are 0.9–2.1 mm in diameter, light yellow to yellow, circular with smooth or rough edges after 2 days of anaerobic incubation at 30 °C on mMRS6 agar. Growth is observed at 4–45 °C, pH 3.5–8.5 and 0–5% NaCl. Strains grow optimally at 30 °C, pH 6.0 and 0% NaCl. Cells are heterofermentative and produce acid from glucose and galactose.

The type strain FUA3913 (=DSM 118650^T^=LMG 33758^T^) was isolated in 2023 from a back-slopped sourdough. The genome size is 1.6 Mb and the G+C content is 37 mol%. The GenBank accession numbers of the sequences of the 16S rRNA gene and the genome are PQ374218 and JBGRWJ000000000, respectively.

## Description of *Levilactobacillus lettrarii* sp. nov.

*Levilactobacillus lettrarii* (let’tra.ri.i. N.L. gen. n. *lettrarii*, named after Silvio Lettrari, who maintains the sourdough from which the bacteria were isolated from).

Cells are Gram-stain positive, non-spore forming, rod-shaped, ~2.2–4.3 µm long and 0.65–0.94 µm wide and occur as pairs or short chains. Colonies are about 4 mm in diameter, flat, irregularly shaped and light beige with rhizoid margins after 2 days of anaerobic incubation at 30 °C on mMRS6 agar plate. Growth is observed at 4–40 °C, pH 4.5–6.5 and at 5% NaCl. Strains grow optimally at 30 °C, pH 6.0 and 0% NaCl. Cells are heterofermentative and all strains metabolize d-xylose, d-galactose, d-glucose, d-fructose, *N*-acetylglucosamine, d-maltose and potassium gluconate. The metabolism of d-ribose and melibiose is strain specific.

The type strain *Lv. lettrarii* FUA3695 (=DSM 118651^T^=LMG 33759^T^) was isolated in 2019 from a back-slopped sourdough. The genome size is 3 Mb and the G+C content is 50.36 mol%. The GenBank accession numbers of the sequences of the 16S rRNA gene and the genome are PQ374219 and CP168709, respectively.

## supplementary material

10.1099/ijsem.0.006726 Uncited Supplementary Material 1.

## Abbreviations

ANI: average nucleotide identity
dDDH: digital DNA–DNA hybridization
MRS: de Man–Rogosa–Sharpe
