# Supplementary material for: Fructilactobacillus frigidiflavus sp. nov., a pigmented species, and Levilactobacillus lettrarii sp. nov., a propionate-producing species isolated from sourdough
Source: Int J Syst Evol Microbiol. 2025 Mar 20;75(3):006726. doi: 10.1099/ijsem.0.006726 (PMC11925284; doi:10.1099/ijsem.0.006726)
Supplement: Uncited Supplementary Material 1. [file ijsem-75-06726-s001.pdf]

Online supplementary material to

***Fructilactobacillus frigidiflavus* sp. nov., a pigmented species, and *Levilactobacillus lettrarii* sp. nov., a propionate producing species isolated from sourdough**

Vi D. Pham and Michael G. Gänzle

University of Alberta, Dept. of Agricultural, Food and Nutritional Science, Edmonton, Canada

Table S1. Sequencing and genome features of *Fructilactobacillus frigidiflavus* FUA3702, FUA3912, FUA3913 and *Levilactobacillus lettrarii* FUA3695, FUA3914.

Organism	FUA3702	FUA3912	FUA3913	FUA3695	FUA3914
Accession number	CP168715	CP168713	JBGRWJ000000000	CP168709	CP168705
Number of contigs	2	2	1	3	3
Contig 1 size (chromosome, bp)	1,626,696	1,618,289	1,626,781	3,028,146	3,028,509
Contig 2 size (plasmid, bp)	3559	11,428	-	28,954	28,953
Contig 3 size (plasmid, bp)	-	-	-	27,339	11,616
G+C content (mol%)	37.00	36.92	37.01	50.36	50.39
Coding sequences	1594	1581	1573	2729	2726
CAZymes	74	73	73	147	145
tRNAs	61	61	61	61	60
rRNAs	27	34	27	18	18
Sequencing coverage (fold)	503	469	310	207	405

Fig. S1. Phylogenetic tree of 16S rRNA gene of the type strains in the genus *Fructilactobacillus* with assembly accession numbers in parentheses. The closely related type strain of *Acetilactobacillus jinshanensis* was used as an outgroup.

Fig. S2. Phylogenetic tree of 16S rRNA genes of the genus *Levilactobacillus* assembly accession numbers in parentheses. The closely related type strain of *Secundilactobacillus collinoides* was used as an outgroup.

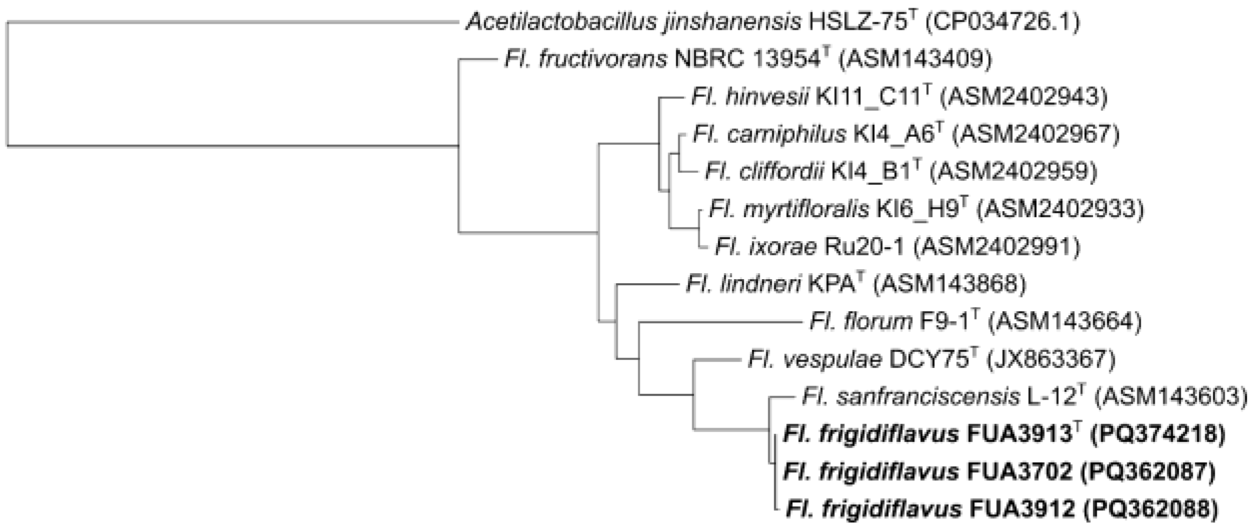
Acetilactobacillus jinshanensis HSLZ-75T (CP034726.1)
Fl. fructivorans NBRC 13954T (ASM143409)
Fl. hinvesii KI11_C11T (ASM2402943)
Fl. carniphilus KI4_A6T (ASM2402967)
Fl. cliffordii KI4_B1T (ASM2402959)
Fl. myrtifloralis KI6_H9T (ASM2402933)
Fl. ixorae Ru20-1 (ASM2402991)
Fl. lindneri KPAT (ASM143868)
Fl. florum F9-1T (ASM143664)
Fl. vespulae DCY75T (JX863367)
Fl. sanfranciscensis L-12T (ASM143603)
Fl. frigidiflavus FUA3913T (PQ374218)
Fl. frigidiflavus FUA3702 (PQ362087)
Fl. frigidiflavus FUA3912 (PQ362088)

Fig. S1.

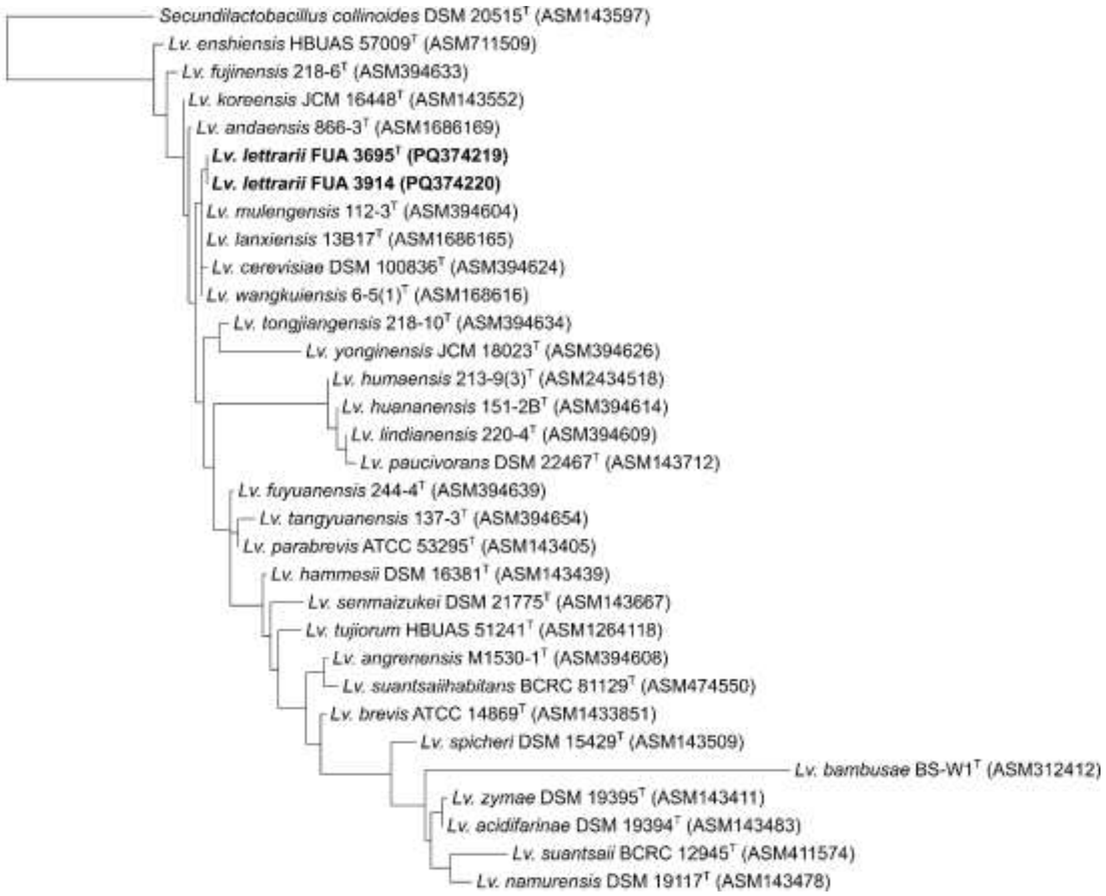
Secundilactobacillus collinoides DSM 20515^{T} (ASM143597)
Lv. enshiensis HBUAS 57009^{T} (ASM711509)
Lv. fujinensis 218-6^{T} (ASM394633)
Lv. koreensis JCM 16448^{T} (ASM143552)
Lv. andaensis 866-3^{T} (ASM1686169)
***Lv. lettrarii* FUA 3695^{T} (PQ374219)**
***Lv. lettrarii* FUA 3914 (PQ374220)**
Lv. mulengensis 112-3^{T} (ASM394604)
Lv. lanxiensis 13B17^{T} (ASM1686165)
Lv. cerevisiae DSM 100836^{T} (ASM394624)
Lv. wangkuiensis 6-5(1)^{T} (ASM168616)
Lv. tongjiangensis 218-10^{T} (ASM394634)
Lv. yonginensis JCM 18023^{T} (ASM394626)
Lv. humaensis 213-9(3)^{T} (ASM2434518)
Lv. huananensis 151-2B^{T} (ASM394614)
Lv. lindianensis 220-4^{T} (ASM394609)
Lv. paucivorans DSM 22467^{T} (ASM143712)
Lv. fuyuanensis 244-4^{T} (ASM394639)
Lv. tangyuanensis 137-3^{T} (ASM394654)
Lv. parabrevis ATCC 53295^{T} (ASM143405)
Lv. hammesii DSM 16381^{T} (ASM143439)
Lv. senmaizukei DSM 21775^{T} (ASM143667)
Lv. tujiorum HBUAS 51241^{T} (ASM1264118)
Lv. angrenensis M1530-1^{T} (ASM394608)
Lv. suantsaiihabitans BCRC 81129^{T} (ASM474550)
Lv. brevis ATCC 14869^{T} (ASM1433851)
Lv. spicheri DSM 15429^{T} (ASM143509)
Lv. bambusae BS-W1^{T} (ASM312412)
Lv. zymae DSM 19395^{T} (ASM143411)
Lv. acidifarinae DSM 19394^{T} (ASM143483)
Lv. suantsaii BCRC 12945^{T} (ASM411574)
Lv. namurensis DSM 19117^{T} (ASM143478)

Fig. S2.
